# Principles and Design of Bionic Hydrogel Adhesives for Skin Wound Treatment

**DOI:** 10.3390/polym16131937

**Published:** 2024-07-06

**Authors:** Chunxiao Wang, Xinyu Zhang, Yinuo Fan, Shuhan Yu, Man Liu, Linhan Feng, Qisen Sun, Panpan Pan

**Affiliations:** 1 Marine College, Shandong University, Weihai 264209, China; wcx17661512175@163.com (C.W.); 17835855549@163.com (X.Z.); 15165524884@163.com (Y.F.); m18214113186@163.com (S.Y.); liuman0725@126.com (M.L.); 18953179141@163.com (L.F.); 13864419248@163.com (Q.S.); 2 National Center for Translational Medicine (Shanghai) SHU Branch, Shanghai 200025, China

**Keywords:** adhesives, hydrogel, natural, bioinspired, wound healing

## Abstract

Over millions of years of evolution, nature has developed a myriad of unique features that have inspired the design of adhesives for wound healing. Bionic hydrogel adhesives, capable of adapting to the dynamic movements of tissues, possess superior biocompatibility and effectively promote the healing of both external and internal wounds. This paper provides a systematic review of the design and principles of these adhesives, focusing on the treatment of skin wounds, and explores the feasibility of incorporating nature-inspired properties into their design. The adhesion mechanisms of bionic adhesives are analyzed from both chemical and physical perspectives. Materials from natural and synthetic polymers commonly used as adhesives are detailed regarding their biocompatibility and degradability. The multifunctional design elements of hydrogel adhesives for skin trauma treatment, such as self-healing, drug release, responsive design, and optimization of mechanical and physical properties, are further explored. The aim is to overcome the limitations of conventional treatments and offer a safer, more effective solution for the application of bionic wound dressings.

## 1. Introduction

The skin, as the body’s largest organ, performs multiple physiological and protective functions, most notably as the first line of defense against external aggressions [1,2,3]. Effective treatment of skin wounds is essential for maintaining this barrier function, not only by ensuring rapid wound closure but also by reducing the risk of infection, accelerating the healing process, and minimizing scar formation [4,5,6]. However, conventional trauma management methods, such as surgical suturing and staple fixation, although widely used in clinical practice, have limitations when dealing with certain vulnerable organs or soft tissue trauma. Specifically, surgical suturing may cause further damage to surrounding delicate tissues, prolong wound healing time, and the removal process of sutures and staples may increase the risk of secondary infections and potentially lead to significant scar formation [7,8,9]. In light of these challenges, medical adhesives, including tissue adhesives, hemostatic agents, and sealants, have emerged as revolutionary wound closure solutions, especially in cases where traditional suturing methods are not applicable or desirable. These adhesives have demonstrated significant advantages in clinical applications, such as the ability to rapidly stop bleeding, reduce the risk of infection, eliminate the need to remove sutures, and reduce postoperative scarring [10,11,12]. Nonetheless, some chemical adhesives currently in widespread use, such as α-cyanoacrylates and polyethylene glycol-based adhesives, despite their excellent adhesion properties, are limited in their application to promote wound healing due to the potential toxicity of their degradation products, inherent toxicity, incompatibility with wetted surfaces, and lack of dynamic adaptability [13,14,15].

In recent years, wet dressings, particularly hydrogel-based dressings, have been demonstrated to expedite the wound healing process by providing an optimal moist healing environment. A moist environment aids in maintaining proper hydration, promoting angiogenesis and collagen synthesis, and facilitating the debridement of necrotic tissue, thereby accelerating the overall healing process [16,17,18]. Hydrogel adhesives, as a three-dimensional cross-linked hydrophilic polymer network with high water content, offer a wide array of potential applications in various biomedical fields due to their excellent biocompatibility, manufacturing versatility, and favorable physical properties, such as their resemblance to the natural matrix. By employing different crosslinking mechanisms and functional group modifications, hydrogel adhesives can achieve diverse crosslinking densities, mechanical properties, and porous structures to cater to the requirements of various medical applications [19,20,21,22]. However, due to the high hydrophilicity and swelling of hydrogel, its adhesion under water is low [23,24].

In response, the development of bionic hydrogel adhesives has received considerable attention from researchers. These materials draw on the adhesion mechanisms of organisms in nature, such as the adhesive proteins secreted by marine mussels and the microstructure of gecko toes, with the aim of mimicking these mechanisms to improve the performance of adhesives [25,26,27]. At the heart of biomimetic technology lies an understanding of the principles of adhesion in nature and the application of these principles to the development of new materials that address the challenges of non-degradability, cytotoxicity, and compatibility with wetted surfaces found in traditional adhesives [28,29,30,31]. By mimicking these natural adhesion strategies and incorporating advances in modern materials science, researchers are working to develop a new class of bionic hydrogel adhesives that not only outperform traditional materials in terms of adhesive performance but are also more compatible with clinical applications in terms of safety and efficiency [32,33].

In this paper, we offer a comprehensive overview of the application of innovative bionic hydrogel adhesives in the field of skin trauma therapy, including their design concepts, principles, and future prospects. We examine the selection and use of natural and synthetic polymers concerning biocompatibility and degradability. By analyzing the adhesion mechanisms of bionic adhesives from both chemical and physical perspectives, this paper further explores the design elements of hydrogel adhesives in skin trauma therapy, such as self-healing, drug release, responsive design, and optimization of mechanical and physical properties, with the goal of overcoming the limitations of traditional therapeutic approaches and providing safer and more effective solutions.

## 2. Adhesion Mechanism of Bionic Hydrogel Adhesives

### 2.1. Direct Extraction and Mimicry of Organismal Chemistry

Life has evolved numerous powerful principles for controlling adhesion, many of which have been applied to engineered materials. Many plants and animals secrete sticky substances to ensure survival and adapt to their environments. Certain functional groups within these compounds interact to establish strong surface affinity and high surface specificity. In this chapter, we will explore natural plant and animal binders based on carbohydrates, proteins, and glycoproteins. We will investigate the components and mechanisms that enable these natural adhesives to function effectively and explore how these principles can be applied to the development of bionic hydrogel adhesives for skin wound treatment.

#### 2.1.1. Protein-Based Natural Plant and Animal Adhesives

The mussel anchors itself to solid surfaces such as rocks and hulls using adhesive proteins (mussel adhesive protein, MAP) secreted by its pedunculated filament glands to withstand wind and wave action. These proteins solidify in seawater to form high-strength, tough, water-resistant fibers. The main adhesion protein, Mefp-1, is enriched with catechol amino acid residues and iron ions, enabling it to rapidly cure and form adhesive structures underwater. A common feature of Mefp proteins is the presence of 3,4-dihydroxyphenylalanine (DOPA), which allows crosslinking of MAP molecules and ensures their curing and excellent water-resistant adhesive properties. DOPA is essential for mussel adhesion and cohesion, forming strong hydrogen bonds with polar polymers and irreversible organometallic complexes with the substrate surface through phenol groups. For example, after the secretion of Mefp-3 and Mefp-5 onto the surface of solid substrates, DOPA reacts with the substrate surface to form a stable complex that firmly adheres the proteins to the surface. Additionally, DOPA in Mefp-2 and Mefp-4 oxidizes to form DOPA quinone, which forms covalent cross-links with lysine and cysteine via a Michael addition reaction or with other DOPA molecules through oxidative coupling. This further enhances protein cohesion. The adhesion mechanism of mussels primarily involves the formation of hydrogen bonds, organometallic complexation, and π–π interactions. The crosslinking reactions between peptides include phenol coupling, Michael addition, and Schiff base substitution. Through these pathways, the mussel enhances adhesion and cohesion in seawater, ensuring it can firmly adhere to various surfaces, making it an effective natural adhesive [34,35,36,37].

The sandcastle worm, known as the ‘mason under the waves’, lives in a mineral shell. Instead of secreting the shell directly, like mollusks, it secretes a gelatinous mucus that binds sand and shell fragments together to form a strong mineral shell. This mucus is produced by the worm’s secretory glands, and each secretory cell contains numerous adhesive particles capable of delivering ‘homogeneous’ or ‘heterogeneous’ particles on demand. The bio-adhesive glue secreted by the sandcastle worm contains moderate amounts of DOPA and includes six different types of adhesion proteins, polysaccharides, and magnesium ions. The proteins responsible for adhesion include Sa-1, Sa-2, Sa-3A, and Sa-3B (each ≤ 22 kDa), differing in their amino acid composition. Sa-1 and Sa-2 are cationic at pH 8.2 and rich in glycine, alanine, tyrosine (functionalized by hydroxylation), and lysine. Sa-3A and Sa-3B are anionic and mainly composed of serine (functionalized by phosphorylation). Tyrosinase and peroxidase in the bio-adhesive link contain DOPA-containing proteins, enhancing cohesion and adhesion. The sandcastle worm’s bio-adhesive undergoes a secondary curing process within a few hours of the initial curing, further enhancing cohesion. Its color gradually changes from off-white to brownish [38,39,40,41]. Eventually, the adhesive cures to form a porous structure filled with interstitial fluid, allowing the sandcastle worm to attach stably to its shell made of sand and shell fragments.

The strong and permanent adhesion of arthropod barnacles differs from that of mussels in that it is achieved by a unique barnacle glue that provides strong and permanent underwater attachment. The barnacle adhesive consists of a variety of adhesive proteins produced by adult glandular cells and secreted through ducts at the interface between the barnacle and the substrate. It undergoes aqueous displacement and solidifies with the surface, thus ensuring that the barnacle is securely attached to a variety of substrates, including metal oxides, glass, plastic, wood, and rock. The main components of barnacle glue are complex biomolecules, approximately 92% of which are proteins, with the remainder being sugars and lipids [42,43]. Kamino et al. proposed a molecular model for the underwater adhesion of barnacle adhesion proteins, with cp19k proteins responsible for adhesion to external substrate surfaces and cp20k proteins responsible for binding to the calcareous base of the barnacle [44]. Despite the low content of lipids and sugars in the barnacle glue, they play an important role in barnacle adhesion to the substrate, and Gohad et al. suggested that barnacle adhesion is a result of the synergistic action of lipids and phosphoproteins. Liang et al. proposed a more refined model of barnacle underwater adhesion based on Kamino’s model, which emphasizes that the barnacle secretes a phase-separating solution during molting that consists of a phenol-rich gel phase and a lipid-rich phase. This mixture removes biofilm from the substrate surface, cleaning it and preparing it for strong adhesion of the barnacle glue. In this way, the barnacle glue achieves strong adhesion to a variety of substrates [45].

#### 2.1.2. Carbohydrate-Based Natural Plant and Animal Adhesives

Polysaccharide and carbohydrate components are also commonly found in natural binders of plant and animal origin, which consist mainly of long chains of straight and branched monosaccharide units bound together by different glycosidic bonds. Their high molecular weight and high density of polar functional groups allow these biopolymers to form unique secondary structures (e.g., helical, laminar, or helical conformations) that enhance the cohesive properties of carbohydrate-based binders [40].

Alginate is a polymer composed of long chains of (1→4)-β-linked D-mannuronic acid (M units) and (1→4)-α-linked L-guluronic acid (G units). This polymer’s molecular configuration allows it to absorb water up to 100 times its own weight and form gels when exposed to divalent cations like Ca^2^⁺. The crosslinking reactions involve the chelation of calcium ions with carboxyl groups on adjacent polymer chains, thereby stabilizing the gel structure [46].

**Figure 1:**
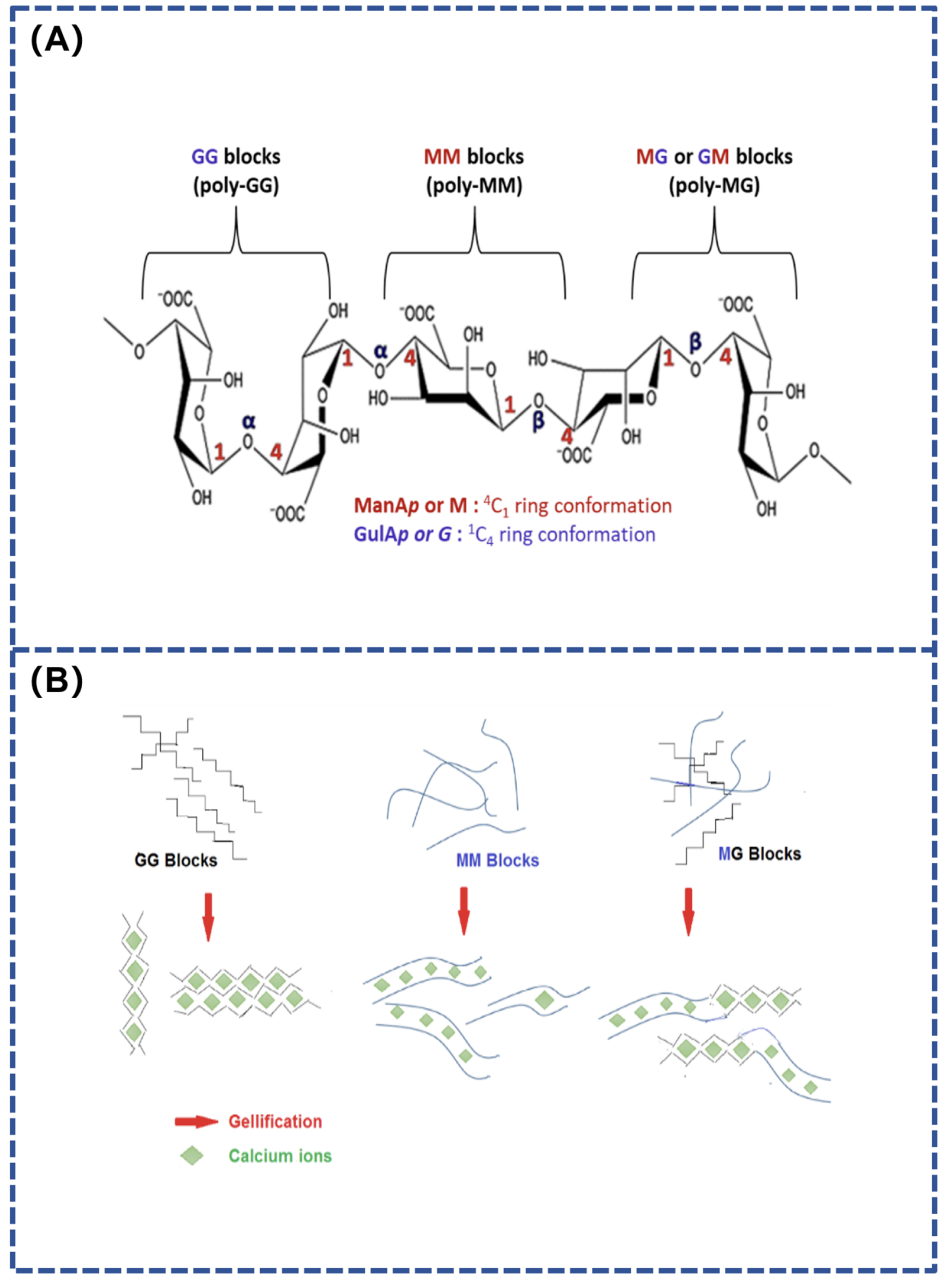
(**A**) Chemical structure of alginate; (**B**) Effect of Ca^2+^ on MG, MM, and GG alginate units [51].

The properties of alginate depend on its viscosity and the ratio of mannuronic and guluronic acids (M/G). High molecular weight increases viscosity, while conformational differences between M and G units result in three chain segments with different properties: MM segments have a linear structure and are elastic; GG segments are helical and rigid; and MG alternating segments are intermediate (Figure 1). Alginate gels with high MM chain segments have good elasticity, while high GG chain segments produce hard, friable, and thermally stable gels. The M/G ratio, sequence, chain length, and molecular weight all affect the properties of the gels, which in turn determine their performance in applications [47,48]. A composite hydrogel adhesives based on sodium periodate-oxidized sodium alginate combined with dihydrazide-modified γ-polyglutamic acid and bio-glass has been developed by Gao et al. This gel was able to achieve strong adhesion to a wide range of tissues and implantable surfaces to promote wound healing [49,50]. On the one hand, the bio-glass provides an alkaline microenvironment to stimulate the binding of oxidized sodium alginate to amino acids in the surrounding tissues, thereby increasing the tissue binding strength. On the other hand, the bio-glass chelates with the carboxyl groups in the oxidized sodium alginate by releasing Ca^2^⁺, thus conferring strong adhesion to the hydrogel adhesives. The remarkable characteristics of alginate, including its biocompatibility, biodegradability, non-toxicity and easy gelation, make it an ideal material for various applications. It can be used in various forms, such as hydrogel and sponge, and has a wide range of applications in the biomedical field.

#### 2.1.3. Glycoprotein-Based Natural Plant and Animal Adhesives

**Figure 2:**
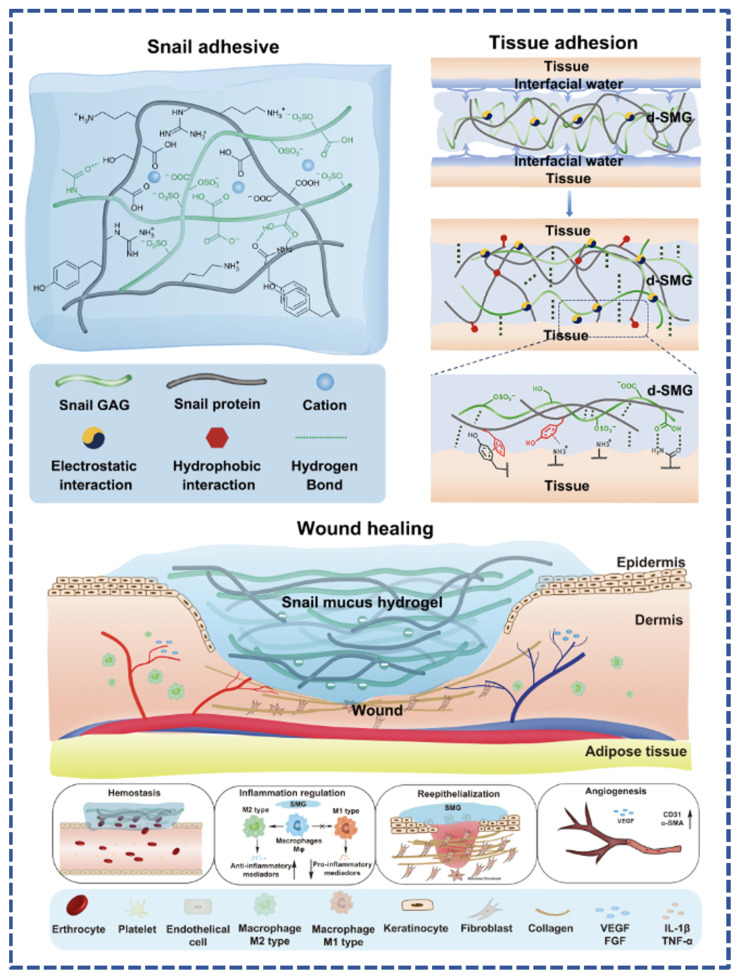
Adhesion mechanism of freeze-dried snail mucus (d-SMG) and its mechanism in promoting wound healing [52].

Tuo et al. collected and freeze-dried natural hydrogel substances secreted by snails, which were sterilized to obtain a porous and high-adhesion performance adhesive (dried Snail Mucus Gel, d-SMG). It was analyzed and found to be mainly composed of biomolecules, such as heparin-like glycosaminoglycans, snail mucin, and snail hemocyanin. Substances with different charges in d-SMG form a unique dual network gel system, which endows the snail mucin gel with strong adhesion and toughness [52]. The adhesive can firmly adhere to wet tissue surfaces with better adhesive strength than fibrin glue commonly used in clinical practice and can effectively bind rat skin incisions with better overall results than sutures and medical 508 glue. d-SMG can also reduce bleeding caused by liver damage and has better hemostatic function (Figure 2). Further studies have shown that this natural bio-adhesive, composed of positively charged proteins and polyanionic glycosaminoglycans, has excellent hemostatic properties, is biocompatible, biodegradable, and significantly accelerates the healing of chronic wounds. d-SMG has remarkable functions, including promoting the transformation of macrophages to anti-inflammatory phenotype, alleviating inflammation of chronic wounds, and promoting epithelial cell regeneration and angiogenesis. Its main active ingredient is rich in heparin-like glycosaminoglycan, which has high affinity and can bind with inflammatory cytokines. The discovery of d-SMG provides a highly important advance in the development of adhesives and dressings.

When ivy climbs objects such as rocks, it secretes tiny particles that cling to the contact surface. These nanoparticles are highly uniform in size and low in viscosity, allowing them to penetrate into the nooks and crannies or cracks of porous surfaces. The researchers found that the arabinogalactan proteins in the nanoparticles play a key role in the subsequent molecular bonding process. As water evaporates, the proteins interact with the pectin and calcium in the ivy secretion to firmly adhere the particles to the surface. After solidifying, this binder is highly tolerant of temperature and environmental changes, allowing ivy to survive natural disasters. Due to ivy’s ability to adapt to a wide range of environmental conditions, its adhesive is considered ideal for the development of protective coatings [53].

The blind eel, which usually lives in deep water up to 100 m below the surface, has an elongated, white appearance and an oval-shaped, sucker-like mouth. When in danger, it secretes natural mucus through glands on both sides of its abdomen, which quickly mixes with water to form a polymer that expands nearly 25,000 times in mass, effectively blocking enemy attacks. From a microstructural point of view, the main components of the mucus are mucin and water, with the mucin being modified by sugar molecules to give the mucus its stickiness. Unlike complex 3D-structured proteins, mucins are usually in the form of long rods, with sugar molecules attached along the protein backbone to form a ‘sugar coating,’ giving mucins excellent water retention and resistance to protein hydrolysis. The concentrated secretion of the blind eel contains discoidal vesicles a few micrometers in size, accompanied by a small number of keratin filaments up to 30 cm in length. After secretion, the structure of keratin filaments changes from α-helical to β-lamellar, forming a stable interconnecting network that attracts large amounts of water and forms hydrogels in the presence of divalent calcium ions [54]. This unique structure provides strong protection for the defense system of the blind eel and offers valuable insights into the development of novel biomedical materials [55].

Adhesive materials, especially underwater adhesive materials, as an advanced functional material have extremely important applications in biomedical fields, such as wound healing. Many kinds of organisms in the marine ecosystem can secrete adhesive proteins with strong underwater adhesion ability, such as mussels, sandcastle worms, barnacles, and blind eels. These organisms provide inspiration and ideas for the development of biomic adhesive materials. Compared with conventional adhesives, marine bio-adhesives are non-toxic, biodegradable, and have strong adhesion. Their most important feature is that they can maintain stable bonding ability under different humidity conditions, which is highly valuable for the development of underwater adhesives.

### 2.2. Mimicking the Physical Structure of Organisms

Bionic hydrogel adhesives can be classified into chemically based biomimetic adhesives and physically based biomimetic adhesives. In this section, we will focus on adhesion designs that mimic the physical structures of organisms, exploring biomimetic strategies and their potential clinical applications. In nature, various structure-related adhesion mechanisms have evolved on bio-adhesive surfaces. Depending on whether the adhesion is derived from interfacial interactions or involves a fluid medium, structure-based bio-adhesion strategies can be primarily categorized into physical adhesion based on interfacial interactions, physical adhesion based on interfacial hydrodynamics, and physical adhesion based on negative pressure adsorption (Table 1).

#### 2.2.1. Physical Adhesion Based on Interfacial Interactions

**Figure 3:**
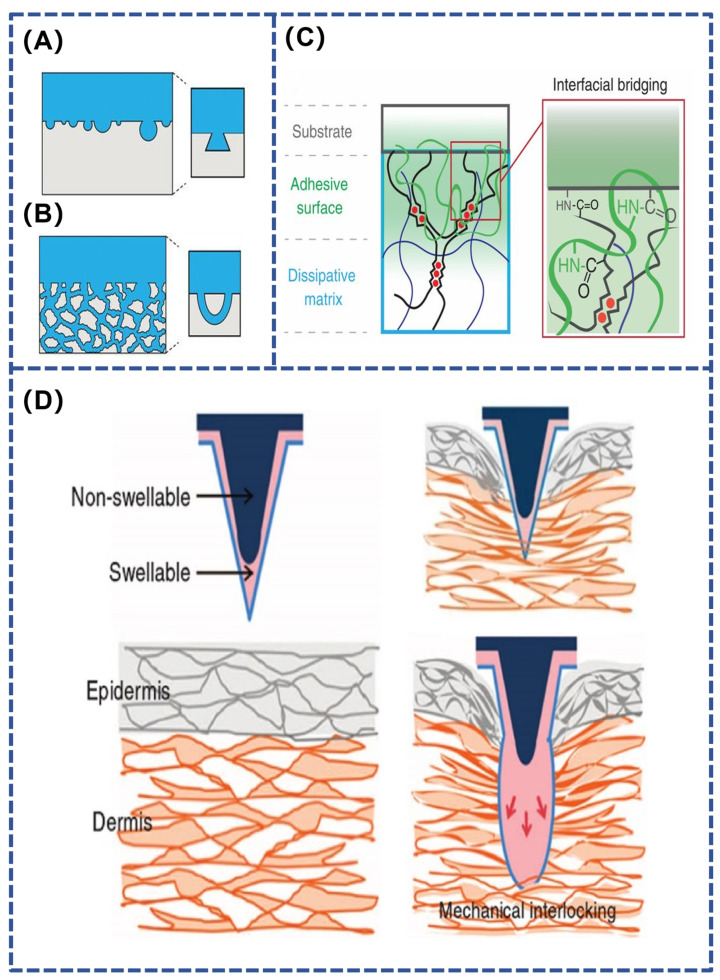
(**A**,**B**) Key-lock and threaded hole topology adhesion structure [57]; (**C**,**D**) Interlocking interface adhesion mechanism [58,62].

Physical adhesion formed by interfacial interactions is achieved through the enhancement of mechanical interlocking or van der Waals forces between interfaces via ordered structures. Mechanical interlocking refers to the formation of adhesion through the surface penetration of the adhesive material, which mechanically interlocks with microscopic holes or irregularities on the surface of the substrate material, creating an interaction similar to that of a lock and key [56]. Mechanical interlocking can be accomplished by geometrically connecting two adherends without the need for either covalent or non-covalent bonding. Mechanical interlocks can be broadly classified into two categories: a lock-and-key topology between adhesive and closed pore adherends (Figure 3A), and a threaded hole topology between adhesive and open pore adherends (Figure 3B) [57]. Mechanical interlocking adhesion between rough surfaces can be enhanced by removing weak surface layers and increasing contact area [58]. Organisms that utilize mechanical interlocking adhesion in nature include planktonic larvae and echinoderms. The microneedle patch, one of the representatives of medical hydrogel adhesives, is inspired by the mechanical interlocking strategy [59,60]. At the molecular scale, polymer molecular chains pass through the adhesion zone and form a molecular interlocking structure on the surface of a highly robust physical gel, which allows the interfacial interaction energy to be transferred to the substrate and dissipated, thus achieving high adhesion strength (Figure 3C) [61]. At the macroscopic level, it is also possible to modify the soluble gel coating on the surface of the microneedle. After insertion into the skin, the hydrogel coating dissolves, forming a mechanical interlocking mechanism (Figure 3D) [62].

#### 2.2.2. Physical Adhesion Based on Interfacial Hydrodynamics

Physical adhesion related to interfacial hydrodynamics is achieved by generating capillary forces or Stefan adhesion with the help of ordered structures [63]. Typical examples include tree frogs, laryngeal discus fish, and beetle flies, whose adhesive organs usually have special micro- and nanoarray structures and corresponding mucus glands. The release of viscous fluids forms many fluid bridges at interfaces containing these special structures, providing adhesion forces based on capillary forces and Stefan adhesion [59].

**Figure 4:**
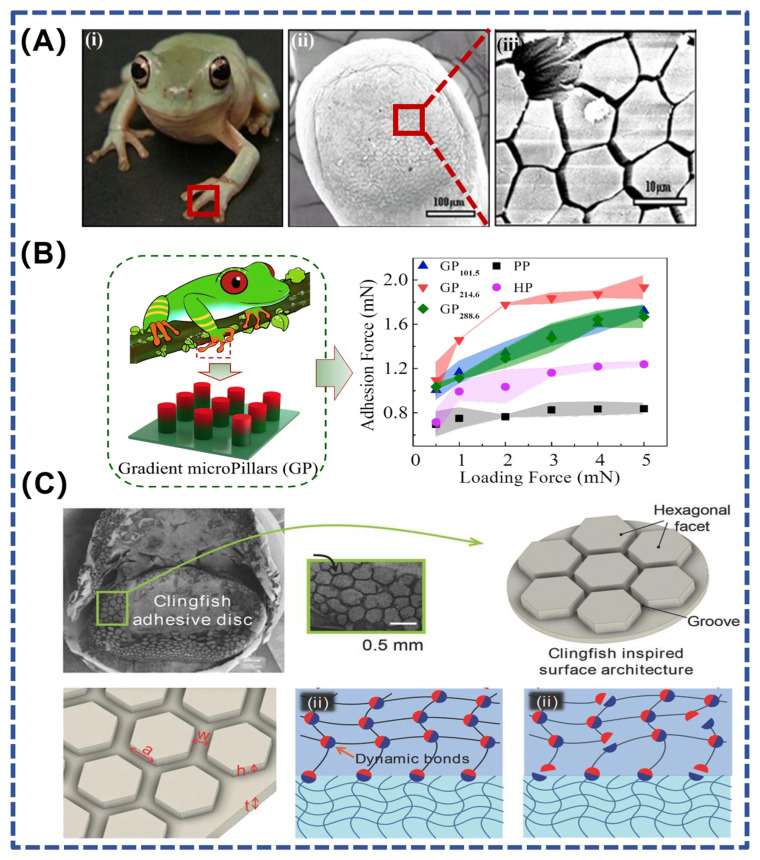
(**A**) Polygonal microstructures on the tree frog’s footpad [63]; (**B**) Gradient composite micropillar array inspired by the tree frog [66]; (**C**) Rapid drainage structure inspired by the clingfish [67]. (i) Tree frog (*Litoria caerulea*) and capillarity on its toe pad at (ii) 100 μm and (iii) 10 μm.

A thin layer of fluid between the pads of the tree frog’s feet and the attached substrate provides good adhesion and friction on soft, wet surfaces, enabling the tree frog to climb flexibly [64]. Tree frog foot pads have protruding polygonal epithelial cells with several micrometer-wide grooves between the polygonal structures (Figure 4A). These channels can drain the liquid at the contact interface, achieving direct contact between solids and thereby providing high adhesion or friction [63,65]. Liu et al. developed a gradient composite micropillar array inspired by tree frogs (Figure 4B), which achieves 2.3-fold dry adhesion and 5.6-fold wet adhesion compared with pure polydimethylsiloxane micropillar arrays [66]. This study also provides a new design strategy for reversible adhesives in robotics, semiconductor processing, and medical engineering.

In addition to this, the laryngeal discus fish has a structure similar to that of tree frogs, allowing it to adhere to rough, wet, or sticky surfaces. By analyzing the ability of the laryngeal discus to rapidly and reversibly adhere to various surfaces underwater, Rao et al. designed hexagonal facets separated by interconnected grooves on the hydrogel surface (Figure 4C). These interconnected surface grooves serve as pathways for the rapid drainage of water during underwater contact. In addition to forming good contact, the discontinuous hexagonal facets improve the compliance of the gel and prevent cracks from continuously expanding across the interface. By combining energy-dissipating hydrogels with dynamic bonds and a surface drainage structure inspired by the laryngeal discus fish, the hydrogel material provides fast, strong, and reversible adhesion underwater [67].

#### 2.2.3. Physical Adhesion Based on Negative Pressure Adsorption

Bionic adhesives that use physical interaction methods to achieve adhesion can include both dry and wet adhesion [68]. Dry adhesion does not allow for firm adhesion in humid environments, so dry bio-adhesives are limited to external applications on human tissues. For example, gecko footpads gain adhesion through their topology, which involves interfacial adhesion across arrays of footpad fibers [69]. These arrays consist of setae with different profiles and spatulate protrusions, where the spatulate protrusions are believed to generate adhesion by van der Waals forces. By mimicking the fibrous structure of gecko footpads and incorporating additional features, the adhesive properties can be extended to a variety of biomedical applications.

Under dry conditions, adhesion relies mainly on van der Waals forces, hydrogen bonding and electrostatic forces. Some terrestrial and marine organisms utilize their natural structures as the fundamental basis for adhesion, employing unique mechanisms (e.g., capillarity, adsorption, and mechanical interlocking) to achieve wet adhesion. Cephalopods, for example, can actively control the grabbing and releasing of a target object by using visual or pressure sensing to learn information about the state and position of the object. Multiple suction cups possessed by cephalopods, such as octopuses, can be independently controlled [70]. The synergy between the independently controllable adhesion devices and the sensing, processing, and control components is key to their ability in flexibly manipulating underwater objects [71].

**Figure 5:**
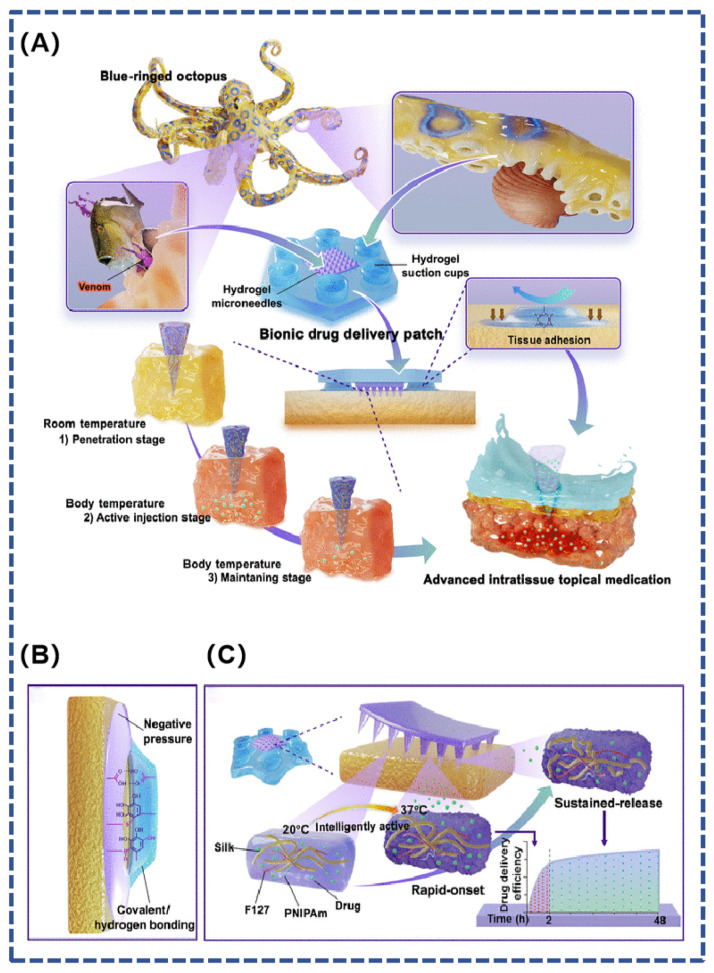
(**A**) A hydrogel microneedle suction cup delivery platform mimicking the blue-ringed octopus, which is able to adhere to wet tissues and autonomously control the multi-stage release of the drug; (**B**) tissue adhesion is achieved by hydrogel chucks by negative pressure and covalent or hydrogen bonds between phenolic hydroxyl groups and tissue proteins; (**C**) composition of bionic hydrogel microneedle suction cups and their effect on drug release [72].

Octopus tentacles are densely distributed with negative pressure suction cups featuring rounded internal protrusions and wrinkled microstructural arrays on the inner bottom edge, which are super-adhesive in both wet and dry environments [26]. Inspired by the super-adhesive capacity of the blue-ringed octopus, Zhu et al. designed a bionic microneedle patch in combination with bio-3D printing technology to achieve controlled topical medication administration in a wet environment (Figure 5). Inspired by the teeth and venom secretion of the blue-ringed octopus, the study prepared gel microneedles that can sense body temperature for active injection to achieve on-demand release of local drugs. The flexible suction cups of the bionic octopus tentacles provide a stable wet bonding effect to ensure the stability of the patch in a wet environment, ultimately enabling controlled and efficient drug delivery to local lesions [72].

**Table 1:** Sources of inspiration and adhesion mechanisms of common bionic hydrogel adhesives based on physical structure.

Biology	Inspiration	Adhesion Mechanism	Ref.
Geckos	Footpad fiber arrays	Gecko footpad arrays consist of hydrophobic setae with different profiles and spatulate protrusions, where the spatulate protrusions generate adhesion through van der Waals forces.	[73]
Tree frog	Tree frog’s footpads	The footpads have protruding polygonal epithelial cells with several micrometer-wide grooves between the polygonal structures, which can drain the liquid at the contact interface, enabling direct contact between solids and achieving high adhesion or friction.	[65]
Insects	Mosquito stinging mouthparts	Serrated microstructure to prepare hydrogel microneedle patches for transdermal drug delivery.	[74]
Sea urchin	Tube feet of sea urchins	The viscoelastic sucker-like tube feet use negative pressure to adhere and promote adhesion. Additionally, the tube feet can regulate adhesion and detachment behaviors by secreting different types of mucus.	[75]
Abalone	Abalone gastropod	The mucus secreted by the epidermis of abalone gastropods is slightly sticky. Combined with the adsorption force generated by negative pressure, this allows the desired attachment state to be maintained. When it is necessary to change position, the abalone moves through wave-like contractions of its foot.	[76]
Octopus	Conical sucker on the carpal foot	The suction cups are primarily composed of funnels. The surface of each funnel features numerous grooves and a finely toothed chitin keratin layer. This unique folded surface enhances adsorption.	[77]
Remora	Back Suction Cup	The vacuum negative pressure suction cup structure consists of an external lip ring and internal fins. Inside the suction cup, continuous pectinate fins can be actively controlled and play an important role in enhancing adhesion.	[78]

## 3. Common Materials for Bionic Hydrogel Adhesives

### 3.1. Natural Polymers

Natural polymers are widely found in plants, animals, and microorganisms and have been widely used in biomedical and other fields due to their many excellent properties. They are commonly used as materials for synthetic hydrogel adhesives. Common natural polymers include chitosan, sodium alginate, hyaluronic acid, cellulose, dextran, xanthan gum, and gelatin. One of the characteristics that these materials share is their structural similarity to biological tissues, making them more easily degradable, which aids in cell growth and tissue healing. These natural polymeric materials are briefly described below (Table 2).

Chitosan is a naturally positively charged polysaccharide polymer composed of glucose units linked by a β-1,4-glucoside bond, and it is commonly extracted from the shells of crustaceans, such as shrimp, crabs, and shellfish. Due to its low toxicity, biocompatibility, antibacterial activity, and mucosal adhesion properties, chitosan has been widely used in biomedical and drug delivery fields [79]. As a natural polysaccharide, its structure is similar to that of polysaccharides in the body, making it less likely to trigger an immune response. The positive charge of chitosan enables it to form electrostatic interactions with other molecules, which also enhances its antibacterial properties [80]. In the body, chitosan is primarily degraded by enzymatic hydrolysis. It is hydrolyzed by a variety of enzymes, including chitinase, chitosanase, and lipase [81]. Once broken down by these enzymes, chitosan is reduced to harmless metabolites that do not cause lasting damage to the organism.

Sodium alginate is a naturally occurring anionic polymer typically obtained from brown seaweeds, such as kelp. It is widely used in the production of hydrogel adhesives due to its biocompatibility, relatively low cost, and mild gelation upon the addition of divalent cations (e.g., Ca^2+^) [47]. However, a major limitation of physical sodium alginate is its very low degradability in the body. When sodium alginate is oxidized, its biocompatibility is greatly improved [82]. Oxidized sodium alginate can be more readily degraded by organisms through hydrolysis reactions, enhancing its suitability for biomedical applications.

Xanthan gum is a natural polysaccharide polymer produced by *Xanthomonas* fermentation, which has good biocompatibility, water solubility, water retention and acid and alkaline resistance [83]. Xanthan gum is non-toxic and does not cause skin irritation or allergies. Its rich hydroxyl and carboxyl groups make it easy to modify by physical, chemical, enzymatic or plasma irradiation treatment to enhance its biomaterial properties [84]. The modified xanthan gum can be combined with other materials, such as polyvinyl alcohol, to form a multifunctional hydrogel adhesive.

**Table 2:** Common natural polymers used for hydrogel adhesives’ formation.

Natural Polymer	Chemical Structures	Preparation	Ref.
Chitosan	A linear polymer chain consisting of β-(1→4) linked 2-amino-2-deoxy-D-glucose units.	It is generally obtained from crustacean shells, such as shrimp, crab, shellfish, etc.	[79]
Sodium alginate	A linear polymer chain consisting of alternating residues of β-D-mannuronic acid and α-L-guluronic acid.	It is generally obtained from brown algae, such as kelp, macroalgae, sargassum, etc.	[51]
Hyaluronic acid	A high molecular weight polysaccharide composed of D-glucuronic acid and N-acetyl-D-glucosamine linked alternately by β-1,3 and β-1,4 glycosidic bonds.	Extracted from animal tissue and fermented with microorganisms using pathogenic and non-pathogenic bacteria.	[85]
Dextran	It is composed of 1,6-linked D-pyranose residues and a few percent of 1,2, 1,3 or 1,4-linked side chains.	It is generally produced by gram-positive, facultative anaerobic cocci and other strains.	[86]
Xanthan gum	It is composed of D-glucose, D-mannose and D-glucuronic acid connected by specific glycosidic bonds.	Produced by fermentation of a strain called *Xanthomonas.*	[83]
Gelatin	It is composed of three α-helical strands, which are connected by hydrogen bonds and other non-covalent bonds to form a triple helix structure.	Generally, it is made from animal bones, raw hides, connective tissues and hard bone tissues, and is obtained by partial hydrolysis and extraction of natural collagen.	[87]

### 3.2. Synthetic Polymers

In addition to natural polymers, synthetic polymers are also widely used as hydrogel adhesives with more controllable structure and stronger properties. Common synthetic polymers include polyethylene glycol, polyacrylic acid, and polyethyleneimine, etc.

Polyethylene glycol is a non-ionic synthetic polymer made by the polymerization of ethylene glycol monomers. Polyethylene glycol is soluble in water and many organic solvents and has good solubility. In addition, it has good biocompatibility, low immunogenicity, and is non-toxic [88]. Polyethylene glycol can be rapidly eliminated from the body without causing damage. The hydroxyl functional groups on the molecular chain of polyethylene glycol can be modified, enhancing the biocompatibility and degradability of polyethylene glycol-based hydrogel adhesives.

Polyacrylic acid is an anionic synthetic polymer formed by the polymerization of acrylic monomers. It has good biocompatibility, high water absorption, non-toxicity, and can be dissolved in water [89]. Polyacrylic acid contains many carboxyl groups, which are highly bioavailable and can be modified using bio-nanomaterials. Polyacrylic acid can be copolymerized with a variety of polymers to form block polymers, and it can also form hydrogen-bonded complexes with various polymers [90]. Due to its excellent properties, polyacrylic acid is widely used in the synthesis of hydrogel adhesives.

Polyethyleneimine is a cationic synthetic polymer, highly alkaline, produced by the polymerization of vinylamine monomers. Polyethyleneimine contains many repeating units of ethylamine and has good water solubility. The molecular toxicity of polyethyleneimine depends on its molecular weight as well as its structure; low molecular weight polyethyleneimine is less toxic and is safer for use in humans and animals, but its cytotoxicity still restricts its application [91]. Polyethyleneimine is a cationic polymer that is able to combine with negatively charged biomolecules, such as drugs and nucleic acids, to form polyelectrolyte complexes through electrostatic action [92]. Polyethyleneimine also has the disadvantage of being non-degradable, but it has been shown that short polyethyleneimine chains can be introduced into longer chains using a linker to obtain biodegradable polyethyleneimine derivatives [93]. The multi-level amino functional groups contained in polyethyleneimine allow it to undergo a variety of chemical modifications to obtain desirable physicochemical properties, and it can be used in the synthesis of hydrogel adhesives.

## 4. Optimal Design of Bionic Hydrogel Adhesives Suitable for Skin Wound Treatment

### 4.1. Self-Healing Property

The role of self-healing hydrogel adhesives is crucial in modern medical applications, especially in the field of wound management. These adhesives are capable of automatically repairing damaged areas and maintaining their structural and functional integrity after damage caused by mechanical forces. The application of self-healing technology not only ensures that the dressing remains intact but also prevents bacterial invasion that may result from damage to the dressing [94]. Additionally, these adhesives maintain peri-wound protection and provide a constant moist environment, creating ideal conditions for cell growth and wound healing. These adhesives adapt to changes in wound shape, increasing patient comfort while reducing the frequency of dressing changes, thereby improving the ease and efficiency of treatment [95]. To further understand how these functions are achieved, the two main design strategies for self-healing hydrogel adhesives are described in detail below:

#### 4.1.1. Design Strategies for Physical Crosslinking

One design strategy for self-healing hydrogel adhesives involves crosslinking through non-covalent bonds, such as hydrogen bonding, metal ion coordination, host-guest interactions, and electrostatic interactions. These interactions enable the material to automatically reconnect broken chains after mechanical damage, rapidly restoring its original function [96]. The self-healing properties of hydrogel adhesives depend on the number and strength of their physical crosslinking interactions. This approach not only quickly restores the hydrogel’s original function but also significantly enhances its mechanical strength.

The power of this mechanism is demonstrated by the interaction of marine bio-catechol groups with metal ions, which is common in bionic adhesives. Yang et al. successfully synthesized a multifunctional hydrogel adhesive using dopamine-functionalized oxidized hyaluronic acid (OD), EPL, and Fe^3+^. The catechol moiety in the hydrogel adhesives provided strong wet adhesion through π–π stacking, metal crosslinking, and hydrogen bonding [97]. At the same time, this multi-crosslinked network improves the mechanical elasticity and cohesion of the hydrogel adhesive, extending the service life and functionality of the dressing. Khodai and his coworkers utilized the reaction of oxidized carrageenan with the formation of a Schiff base from dopamine, adding Zn^2+^ with metal-catechol complexation of the catechol moiety to prepare antimicrobial and self-healing hydrogel adhesives. Alternating strain confirmed its self-healing ability through the reconstruction of imines and other non-covalent bonds in hydrogel adhesives [98].

Hydrogen bond crosslinking is a common synthetic strategy for self-healing hydrogel adhesives because hydrogen bonds are widely present in natural and synthetic compounds. Hydrogen bonds are formed by electrostatic interactions between hydrogen atoms and electronegative atoms, with a bond energy of about 42 kJ·mol⁻^1^,and their formation and fracture speed is very fast. For example, Guo et al. developed antioxidant, anti-inflammatory, and antimicrobial tannin-crosslinked mussel mimetic medical adhesives by utilizing the hydrogen bonding interactions between natural plant polyphenol tannins (TA) and citrate-based mussel-inspired adhesive prepolymers capable of instantaneous (<25 s) gel formation. Rheological tests confirmed the self-healing ability of the adhesive, with its storage modulus (G’) mostly recovering even after four cycles of large strain (100%) for one minute, followed by one minute of relaxation [99]. Additionally, Dai et al. designed a collagen-based tissue adhesive with rapid removal of interfacial water and multiple dynamically reversible osmotic cross-links by simulating key processes in adhesives secreted by mussels and oysters. Rheological test confirmed the self-healing behavior. With the increase of shear strain from 1% to 500%, the hydrogel network broke. However, when a low strain of 1% is applied, the loss modulus and storage modulus quickly return to their initial values. This combination of collagen and starch depends on reversible hydrogen bonding, dynamic coordination and electrostatic interaction, showing excellent self-healing performance and dynamic coordination [100]. Professor Bruce P. Lee’s group developed a composite gel tissue adhesive with remarkable plasticity and adhesive properties, showing potential for biomedical applications. The innovative material consists of two key components: a dopamine-capped, multi-armed polyethylene glycol (PEG-D) and lithium saponite [101]. These two components rapidly interact to form a highly viscous, malleable composite gel. Once applied to a wound, the dopamine portion stabilizes the gel’s morphology by forming a covalent crosslinking structure. Additionally, the higher number of PEG-D arms and the content of lithium saponite significantly increase the number of physical crosslinking sites, where hydrogen bonding and dispersion forces are the main forces. The gels exhibited excellent plasticity, rapidly recovering their original energy storage modulus after a day of exposure to 1000% shear once the shear force was removed. This property demonstrates the ability of PEG-D and lithium saponite nanoparticles to rapidly re-establish physical crosslinking points, highlighting their potential in medical adhesive design. Xiao et al. achieved the desired self-healing and stretchable properties of frequently moving joints by blending polyacrylic acid copolymers, isopentenyl polyethylene glycol ethers and tannic acid (TA) into hydrogel adhesive with strong wet adhesion. The PEGylated chain segment on the polyacrylic acid prevents electrostatic repulsion between the ionized carboxylate group and the adsorbed TA, thus forming a bonding layer, and the structure recovers quickly even after five alternating repeated load stress cycles.

#### 4.1.2. Design Strategies for Chemical Crosslinking

Another strategy is to design self-healing hydrogel adhesives through reversible chemical crosslinking, utilizing mechanisms such as the Diels–Alder (DA) reaction, borate bonding, hydrazone bonding, and Schiff base reaction. The core advantage of the reversibility of these chemical reactions is that they allow the material to automatically rebuild its chemical structure after damage without external intervention. Taking the Diels–Alder reaction as an example, this reaction is widely used in the preparation of self-healing urethanes due to its thermal reversibility, mild reaction conditions, and low degree of side reactions. Specifically, the DA reaction allows the conjugated diene and unsaturated carbon-carbon double bonds of the polyurethane molecular chain to reorganize after damage via a 1,4-addition reaction, leading to self-healing on a macroscopic scale. When driven by heat, the DA bonds in polyurethanes can break in the reverse direction to form short molecular chains, which are able to bridge the cracks quickly due to their high mobility. These chains then rejoin and revert to long chains when the temperature drops slightly, achieving self-healing. In addition, the mobility of the DA-bonded polyurethane molecular chain segments significantly affects their self-healing ability. At a certain temperature, the higher mobility of the molecular chain segments increases the probability of DA bond encounters, thus enhancing the self-healing rate. The addition of ionic liquids (EMITFS) as plasticizers can further improve the mobility of the chain segments and effectively reduce the temperature required for self-healing [102]. Hydrogel adhesives based on the DA reaction can self-heal at room temperature and are suitable for long-lasting skin-fitting applications. In their study, Liu et al. selected deferoxamine (DFO) as the backbone and used polyethylene glycol (PEG) as a crosslinker to modify hyaluronic acid (HA) to prepare a biocompatible material. They added imidazole polymer (ionic liquids) (PILs) to the modified HA/PEG precursors through a DA click reaction to prepare a hydrogel adhesive with excellent self-healing properties and mechanical properties [103].

In addition, self-healing hydrogel adhesives can be synthesized using borate ester bonds. Boron esters and borate esters are pentagonal or hexagonal rings in which the boronic acid is bonded to cis-1,2- or 1,3-diols. Specifically, in environments where the pH is higher than the pKa of the boronic acid-based materials, these polymers form hydrogel adhesives by crosslinking with the diols and can reach a dynamic equilibrium between the boric acid and the diols depending on the pH. The non-rigid nature of the borate bond allows it to interact with the surrounding free diols and boric acid during hydrolysis to form new crosslinking sites. Thus, depending on the pH or the heat-sensitive aqueous medium, the borate bonds in hydrogel adhesives can exhibit self-healing properties in the absence of external stimuli [104]. This self-healing ability is particularly significant for hydrogels composed of rigid nanofibrillar cellulose and polyvinyl alcohol (PVA) networks, which are synthesized with borax as a crosslinking agent. These hydrogels are capable of rapidly eliminating the interface upon contact and self-healing to restore the hydrogel’s ionic conductivity and mechanical properties within a short period of time. In the absence of an external force, the interfaces of the two types of hydrogels disappear after 5 min of contact. The ionic conductivity and mechanical properties of the hydrogels were restored by 89.8% and 93.0%, respectively, after healing. The healing rate remained at 83.0% after five repetitions [105]. In addition, the hyaluronic acid hydrogel based on the phenylboronic acid–diol ester bond developed by Gu’s team shows excellent self-healing performance, injectability, and compressive strength through rheological and compressive tests, which proves the application potential of this kind of hydrogel in the biomedical field [106].

Schiff base bonds are formed by the reaction of reactive carbonyl groups with amino compounds. This process of forming imine or alkyl-imine structures ensures the self-healing ability of hydrogels and is widely used in their synthesis. For example, the formation of oxidized sodium alginate (OSA) and carboxymethyl chitosan (CMCS) Schiff base bond combined with the free radical polymerization of acrylamide (AM) monomer forms a unique multi-network structure, which makes the hydrogel exhibit excellent mechanical properties and self-healing ability in flexible open wounds [107]. In addition, acyl-hydrazone bonds are formed by the condensation reaction of hydrazine with carbonyl groups (such as aldehydes or ketones). These bonds have also been used to construct self-healing hydrogel adhesives. Although acyl-hydrazone bonds are similar to Schiff base bonds, they are more reactive and more stable in water. For example, the hydrogel adhesive developed by using the catalyst-free crosslinking reaction between phthalaldehyde and amine (hydrazines) can quickly close wounds and promote healing, and the effect is better than that of commercially available fibrin glue and cyanoacrylate glue [108].

With these two main design strategies, self-healing hydrogel adhesives exhibit significant advantages in wound healing applications. However, single chemically or physically crosslinked hydrogel adhesives have limitations in aspects such as elasticity, tensile properties, and self-healing rates. Specifically, although single chemically crosslinked hydrogels have high rigidity and toughness, they often lack sufficient stretchability and elasticity for practical applications and have slow self-healing rates due to the absence of an effective energy dissipation mechanism. Physically crosslinked hydrogels are easy to handle but often have poor mechanical properties, making them inadequate for medical applications [109]. In contrast, the synergistic effect of using multiple dynamic bonds not only enhances the overall performance of the hydrogel but also improves its self-healing rate, allowing the hydrogel to rapidly recover its structure and function without external intervention. This multi-dynamic bonding strategy has gradually become a mainstream trend in the preparation of self-healing hydrogel adhesives [110,111]. By combining different types of reversible bonds, these hydrogels can quickly respond to physical damage and automatically repair fractures or damages while maintaining excellent mechanical properties. Furthermore, this design approach not only enhances treatment efficacy and reduces the risk of infection but also lowers overall treatment costs due to less frequent replacement. Going forward, research will likely focus on further enhancing the self-healing speed and efficiency of these hydrogels. Overall, through these studies, it is expected that more powerful self-healing materials with a wider range of applications will be developed to provide more effective treatment options in the clinic.

### 4.2. Drug Release and Biological Activity

In the field of wound therapy, bionic hydrogel adhesives have been endowed with excellent skin repair capabilities by mimicking the key structures and chemical compositions of natural plants and animals. However, wound healing is a highly complex cascade process that involves the spatially and temporally synchronized collaboration of various cell types during the hemostasis, inflammation, growth, re-epithelialization, and remodeling phases. The complexity and variability of the wound environment mean that simply mimicking natural mechanisms is not sufficient to meet all the challenges. For this reason, a variety of therapeutic components have been incorporated into bionic hydrogel adhesives to enable them to play key roles in the various stages of wound healing. These components include hemostatic factors to control bleeding, antimicrobial agents to effectively fight infection, bioactive substances to promote cell regeneration and tissue repair, and intelligent systems to precisely regulate drug release. The combined use of these design elements significantly enhances the therapeutic efficiency and effectiveness of bionic hydrogel adhesives in wound management, while broadening their scope of application in promoting wound healing.

**Figure 6:**
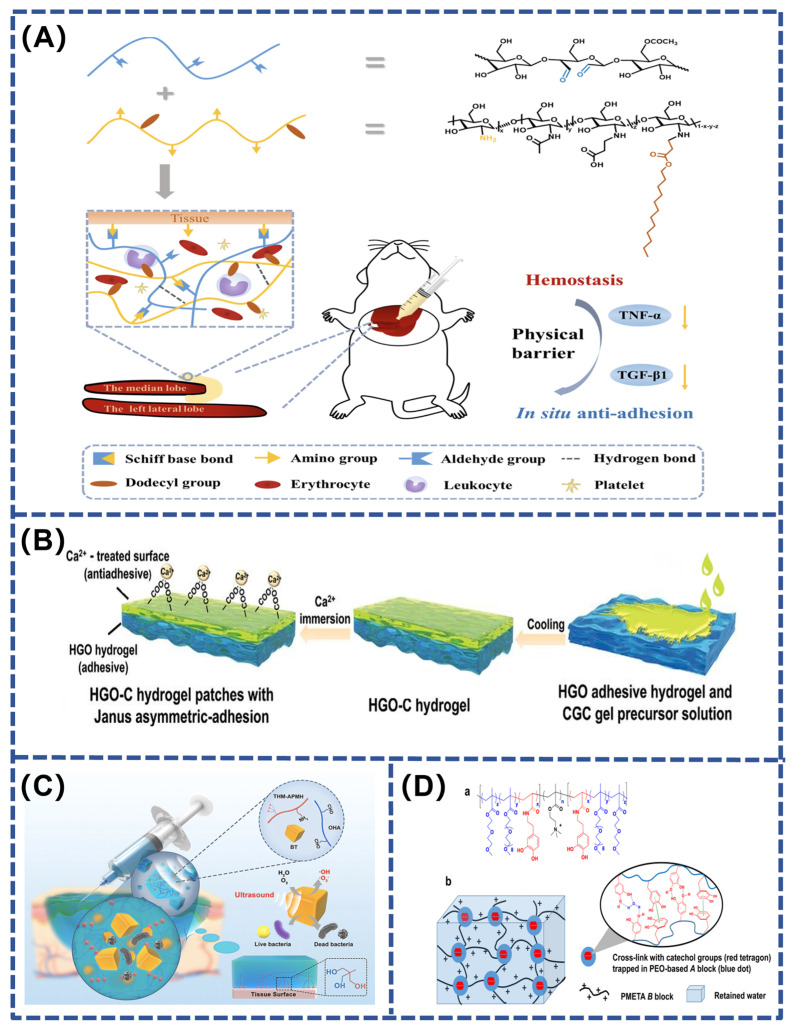
(**A**) Schematic diagram of injectable hydrogel adhesives [114]; (**B**) schematic diagram of the preparation process of the Janus asymmetric adhesive HGO–C hydrogel patch [115]; (**C**) synergistic effect of the BT–OHA/THM–APMH hydrogel and ultrasound-triggered piezo-catalytic therapy in repairing infected wounds [116]; (**D**) diagram showing hydrogel adhesives structure and mussel-inspired self-healing mechanism [117].

Bleeding after injury is often unavoidable, making hemostasis one of the key steps in wound management. The development of bionic hydrogel adhesives effectively promotes hemostasis and prevents the reopening of wounds through their unique bio-adhesive function and ability to absorb wound exudates. In the hemostatic application of hydrogels, materials with hemostatic properties are usually added to enhance their performance. Examples include chitosan, gelatin, and collagen—natural polymerizations that not only accelerate the clotting process but also provide protection to wounds and support their rapid healing by forming a stable cover layer [109,112,113]. In addition, silicon-based inorganic hemostatic materials (kaolin, zeolite, etc.), which have mesoporous structures that can absorb blood and promote coagulation, and metallic hemostatic materials, commonly Ca^2+^ ions that accelerate thrombosis by promoting the conversion of prothrombin to thrombin, can further enhance the hemostatic effect. The use of these hemostatic materials not only enhances the functionality of the hydrogel but also brings a more efficient and safer solution for wound treatment. A specially designed hydrogel contains thrombin concentrate (polymerization inducer) and fibrinogen (coagulation component), a combination that not only rapidly stops bleeding but also forms a stable overlay on the wound surface and cures rapidly. This design optimizes the hemostatic function of the hydrogel while providing effective protection and support to the wound, thus promoting a faster healing process. The hydrogel developed by Wei et al. used oxidized konjac glucomannan (OKGM) and dodecyl modified N-carboxyethyl chitosan (DCEC). This hydrogel was prepared by a Schiff base reaction and showed excellent hemostatic effects and adhesion properties. The introduction of DCEC made the hydrogel not only hemostatic but also antimicrobial. However, DCEC–OKGM hydrogels have anti-adhesion properties, which are attributed to their formation of a physical barrier between damaged tissues and adjacent organs (Figure 6A). This barrier action prevents direct adhesions caused by cell proliferation and migration during wound healing and inhibits tumor necrosis factor (TNF) expression [114]. Another innovative approach was proposed by Zhang’s team, who constructed a multifunctional hydrogel patch with Janus asymmetric adhesion, combining the properties of both adhesive and non-adhesive hydrogels. This design allows the adhesive hydrogel portion to firmly adhere to the wound through hydrogen bonding and electrostatic interactions, rapidly stopping bleeding and promoting healing, while the non-adhesive portion acts as a physical barrier to protect the wound from external contamination (Figure 6B). This asymmetric design not only enhances hemostasis but also optimizes the efficiency and safety of hydrogel application in clinical treatment [115].

As the primary line of defense between the body and the external environment, the skin plays a key role in preventing microbial invasion. When the skin’s intrinsic protective mechanisms are compromised, wounds are highly susceptible to bacterial infection, which may slow down the healing process. Traditionally, antibiotics are added to hydrogel excipients to confer bactericidal capacity, but this approach may lead to problems such as uncontrolled drug release and multi-drug resistance caused by antibiotic overuse. Therefore, in recent years, researchers have sought non-antibiotic antimicrobial strategies to overcome these limitations. These new strategies include the use of metal ions and non-traditional antimicrobial agents such as silver (in the form of silver nanoparticles and silver nitrate) and zinc. These metal ions exert bactericidal effects by interfering with bacterial proteins through complexation. Although using metal ions to kill bacteria is a well-established area of research, there are concerns about their use in the body due to potential toxicity and allergic reactions (hypersensitivity).

Photothermal therapy (PTT) is an attractive and promising method of localized heating. The main photothermal agents now used to prepare NIR-responsive photothermal agents include transition metal sulfide–oxide nanomaterials (e.g., CuS, TiO_2_, and MnO_2_), metal nanostructures (e.g., Cu NPs and Au NPs), carbon-based materials (e.g., carbon nanotubes and graphene and graphene oxide), conjugated polymers (e.g., poly-pyrrole, PDA, and polyaniline), iron–catechol complexes, and Prussian blue nanoparticles. Zhao et al. developed a photothermally active dual network hydrogel for treating wounds infected with multi-drug-resistant bacteria. It is photothermally responsive through catechol–iron ion metal coordination, which raises the hydrogel temperature during near-infrared irradiation, thereby killing the bacteria at the wound site and protecting the wound from infection. In addition, metformin-containing CuPDA NPs composite hydrogels (Met@CuPDA NPs/HG) were prepared by dynamically combining dopamine-modified gelatin (Gel-DA), Cu-loaded polydopamine nanoparticles (CuPDA NPs), and phenyl borate-modified hyaluronic acid (HA-PBA). These hydrogels not only exhibit an excellent photothermal effect but also slowly release Cu^2+^ to protect the wound from infection for a long time and effectively reduce inflammation, showing great potential for treating chronic diseases, such as diabetes mellitus [118]. Another innovative strategy is ultrasound-triggered piezo-catalytic therapy, such as the multifunctional hydrogel designed by Liu et al. Its loaded barium titanate nanoparticles exhibit excellent antimicrobial effects due to a strong inbuilt electric field, which rapidly generates reactive oxygen species (ROS) under ultrasonic vibration (Figure 6C). This significantly improves the biosafety of the treatment compared to conventional photodynamic therapy and shows great potential for harmless treatment of bacterial infections [116]. Additionally, the catechin-functionalized polyethylene glycol hydrogel is self-repairing and has excellent thermal sensitivity, effectively inhibiting the growth of *E. coli* (killing efficiency > 99.8%) and preventing cell adhesion. It also autonomously heals from repetitive damage through mussel-inspired catechol-mediated hydrogen bonding and aromatic interactions. Importantly, the material is metal-free and therefore has low cytotoxicity (Figure 6D). Overall, these findings demonstrate that polyethylene glycol (PEG) has great potential for bioengineering applications [117]. Furthermore, inspired by the natural extracellular matrix (ECM), Liu et al. coupled glucomannan with endogenous antimicrobial peptides via dynamic, acid-sensitive imine bonds, and hyaluronic acid (HA) grafted with collagen tripeptide (GPHyp). This combination developed an ECM-mimicking hydrogel that provides structural support for the adhesion, migration, and proliferation of cells, as well as the essential amino acids for collagen reconstruction. The ability to remodel damaged tissues, reduce inflammation, and promote angiogenesis without the need for additional drugs and exogenous cytokines provides an effective therapeutic strategy for chronic wounds, especially Methicillin-resistant *Staphylococcus aureus* (MRSA)-infected diabetic and burned skin [119].

In traditional drug delivery systems, instability in drug concentration and large fluctuations in active drug levels often affect therapeutic efficacy. To address these problems, controlled drug delivery systems (DDS) have been developed to improve drug utilization efficiency and effectiveness while reducing costs and side effects. Smart/stimuli-responsive hydrogels show great potential in this field due to their high water content, sensitivity, and adjustable structure. These hydrogels can be loaded with bioactive ingredients and react to specific signals (such as pH, temperature, and light) to release drugs or activate bio-adhesives [120,121,122,123,124]. They can quickly respond to environmental stimuli by swelling, contracting, or undergoing sol-gel phase transitions, thus promoting wound healing. In addition, hydrogel adhesives can stop bleeding, seal wounds, maintain tissue function, and provide antibacterial protection, making them a key area of research and development. The selection of raw materials and the loading of bioactive substances (such as cells and drugs) are critical factors affecting their effectiveness. In the future, the application of hydrogel adhesives may become more personalized and diversified, incorporating features such as electrical conductivity for electrically active tissues and contractility to assist in wound closure. These advancements highlight the potential of hydrogel adhesives in improving therapeutic outcomes and patient rehabilitation.

### 4.3. Stimulus–Response Design

Wound healing involves complex changes influenced by factors like temperature, pH, oxygen, and glucose levels. Conventional hydrogel dressings often fail to meet the needs of complex wounds. Stimuli-responsive hydrogel adhesives can actively respond to the wound environment by sensing and adjusting to these factors. For example, temperature-sensitive hydrogel adhesives can change their physical properties and release built-in drugs when body temperature rises to inflammatory levels, while pH-sensitive hydrogel adhesives can adjust their solubility or structure to adapt to different stages of the healing process when the wound environment shifts from acidic to alkaline. In addition, ROS-responsive hydrogel adhesives can trigger drug release when local ROS concentrations increase, effectively responding to oxidative stress. For specific diseases such as diabetes, it is particularly important to develop hydrogel adhesives that are responsive to changes in glucose levels because of persistently high blood glucose levels and accompanying impaired healing. These hydrogel adhesives can modulate drug release in response to changes in blood glucose, thereby optimizing therapeutic efficacy [125,126]. This intelligent adjustment promotes effective healing, reduces chronic wound recurrence, and alleviates patient pain and financial burden, offering a personalized and targeted therapeutic strategy for future wound treatments.

**Figure 7:**
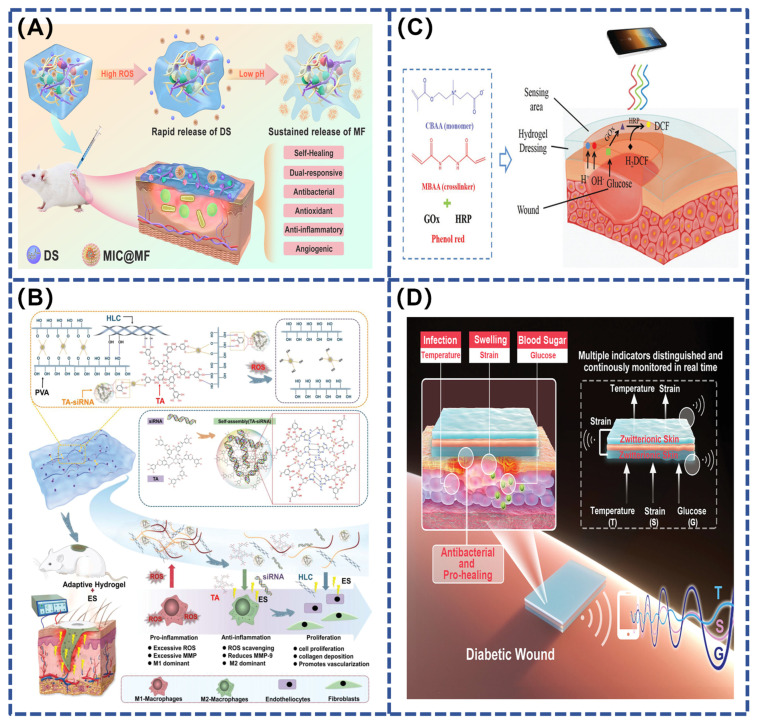
(**A**) Schematic diagram of the manufacturing process for embedding DS and MIC@MF into POD/CE hydrogel [127]; (**B**) repairing chronic diabetic wounds using ES therapy and adaptive conductive PHTB (TA-siRNA) hydrogel [128]; (**C**) scheme for detecting the pH and glucose concentration of wound exudate using PCB hydrogel dressing [130]; (**D**) schematic diagram of a sandwich-structure sensor based on a multi-responsive zwitterionic epidermis [131].

Stimuli-responsive hydrogel adhesives are becoming increasingly important in the field of wound therapy, especially in the management of chronic and heavily infected diabetic wounds. Diabetic wounds are complex environments that are difficult to treat. The complexity of these wounds stems from their unique microenvironment, including persistent hyperglycemia, low local pH, and excess reactive oxygen species (ROS), which may lead to persistent inflammation, abnormal vascular endothelial function, and tissue necrosis, collectively slowing down the natural healing process. Single stimulus-responsive hydrogel adhesives, although capable of responding to specific physiological changes, often struggle to achieve efficient and precise targeted drug release, especially in variable wound environments(Table 3). Therefore, the development of composite stimulus-responsive hydrogel adhesives that can simultaneously respond to multiple physiological signals is particularly critical. For example, Kong et al. prepared an injectable glycopeptide hydrogel adhesive with pH and ROS responsiveness using phenyl-boric acid-grafted glucan oxide (POD) and caffeic acid-grafted ε-poly-lysine (CE) materials (Figure 7A). The adhesive was cross-linked via dynamic borate and Schiff base bonds and loaded with diclofenac sodium (DS) with anti-inflammatory properties and pH-responsive micelles (MICs) containing mangiferin (MF) that promote angiogenesis. In the process of wound healing of diabetic infection, with the break of borate and Schiff base bonds sensitive to acidic and oxidizing environment, antibacterial substances CE and anti-inflammatory substances DS and MIC are released, effectively inhibiting inflammation and antibacterial. Subsequently, the continuous acidic environment gradually releases MF, promoting neovascular formation and accelerating chronic wound healing [127]. Similarly, Fan et al. developed a novel TA–siRNA nanogel based on the self-assembling interaction of tannic acid (TA) with short interfering RNA (siRNA). This nanogel, combined with polyvinyl alcohol (PVA), human-like collagen (HLC), TA, and borax crosslinking, forms an adaptive and conductive PHTB hydrogel. It is designed to maximize therapeutic effects by responding to stimuli, such as ROS and electrical stimulation (ES). In a high ROS environment, the hydrogel’s borate bond breaks, releasing TA-siRNA nanogels that reduce matrix metalloproteinase-9 (MMP-9) expression, while TA and HLC promote collagen expression and reduce inflammation (Figure 7B). Electrical stimulation further enhances the release and endocytosis of TA-siRNA nanogel, activates macrophage polarization and neointimal formation, and accelerates diabetic wound healing [128].

In the field of diabetic wound therapy, multi-stimulus responsive hydrogel adhesives have demonstrated significant benefits in terms of their ability to rapidly respond to environmental changes and release therapeutic agents at the right time. These hydrogel adhesives adapt to dynamic changes in the wound microenvironment, thereby improving the relevance and efficacy of the treatment [129]. In combination with sensing technology, the range of applications and functionality of these hydrogel adhesives is further extended. By integrating sensors, these hydrogel adhesives can not only serve as carriers of therapeutic agents but also monitor the state of the wound in real-time, such as changes in temperature, stress, and biochemical markers, providing richer data support for treatment, and thus offering a more comprehensive treatment plan.

For example, Zhu et al. developed a zwitterionic hydrogel for monitoring pH and glucose concentration in diabetic wounds. This hydrogel contains phenol red dye, which is sensitive to pH changes, and encapsulates glucose oxidase (GOx) and horseradish peroxidase (HRP). These enzymes catalyze the oxidation of glucose to produce fluorescent products. Due to the hydrogel’s anti-biofouling properties, the immobilized enzymes maintain high activity and stability when treating wound exudates, whereas free enzymes are unstable. For ease of use, color and fluorescence signals can be accurately measured via a spectrophotometer or smartphone (Figure 7C). Clinical trials have shown that the hydrogel not only provides better monitoring results but also significantly accelerates the healing of diabetic wounds compared to DuoDerm dressings [130]. In contrast, Zhang’s team expanded the sensing technique to synthesize a multi-stimulation-responsive conductive hydrogel (SB–N–MB) composed of sulfonated betaine (SBMA), n-isopropylacrylamide (NIPAAm), and methyl-acrylamide phenyl-boric acid (MPBA).They further developed an ion skin sensing system with a ‘sandwich’ structure that is able to monitor temperature, strain and glucose levels in real-time and effectively distinguish between these signals. The layered design of the system ensures precise transmission of temperature changes (inflammation), strain changes (wound swelling) and glucose concentration changes (blood glucose) without signal interference (Figure 7D).This sensor-integrated hydrogel system not only improves the efficiency of local drug delivery, but also helps health care workers better understand microenvironmental changes during wound healing by continuously monitoring key physiological indicators [131].

Stimulus–responsive hydrogel adhesives are crucial for treating chronic refractory wounds. By regulating drug release and dressing performance, they can effectively respond to changes in the wound microenvironment, thus reducing wound recurrence, pain, and the economic burden on patients [132,133]. Combined with sensing technology, hydrogel adhesives demonstrate potential for precision treatment and personalized medicine, providing a new direction for future wound care.

**Table 3:** Applications of bionic hydrogel adhesives for various wound types.

Wound Type	Characteristics	Bionic Hydrogel Adhesives Applications	Ref.
Full-thickness skin defects	Clean, controlled environment	Infection prevention, scar minimization	[134]
Burns	Acute, high risk of infection, excessive tissue fluid exudation, tissue necrosis, hypoxia–ischemia, and decreased immunity	Antibacterial, pain relief, intelligent delivery of stem cells, growth factors, etc.	[135]
Diabetic wounds	Chronic, excessive chronic inflammatory response, impaired angiogenesis, excessive oxidative stress, and hypoxia in the wound environment	Adhesion, self-healing, intelligent response release, ROS scavenging and oxygen production	[136,137]

### 4.4. Regulation of Mechanical and Physical Properties

Improving the mechanical and adhesion properties of bionic hydrogel adhesives is crucial for skin wound healing. These properties not only directly impact the functional effectiveness of hydrogel adhesives but are also key factors in facilitating wound closure, reducing the risk of infection, and accelerating the healing process [138,139]. Specifically, the mechanical properties of hydrogel adhesives, especially their strength and elasticity, ensure that they can withstand the physical stresses in the wound area and prevent fracture or deformation, thus maintaining the wound in a stable state. At the same time, the excellent adhesion properties of the hydrogel adhesives ensure that they fit tightly to the wound, forming an effective barrier to isolate it from the external environment and prevent microbial invasion and other possible infection factors [136,140]. In addition, adhesion facilitates the hydrogel’s interaction with cells on the wound surface, supporting cell migration and new tissue production, which are important steps in wound healing.

To further enhance these properties, several commonly used methods include dual reticulated hydrogel adhesives, dual physicochemical crosslinked hydrogel adhesives, and the construction of nanocomposite hydrogel adhesives. Dual reticulated hydrogel adhesives technology improves the mechanical strength of the overall structure by combining two polymer networks: a stiff but brittle polymer and a flexible, ductile polymer that interact with each other. Dual physicochemical crosslinking hydrogel adhesives, on the other hand, not only enhance the tensile strength and toughness of the hydrogel adhesives through a dual mechanism of chemical and physical crosslinking but also ensure its durability and stability under extreme conditions. Nanocomposite hydrogel adhesives utilize nanomaterials, such as inorganic salt particles or metallic nanomaterials, as crosslinking points, which enhance the network structure and allow some of the polymer chains to break and consume energy under stress, thereby increasing overall elongation and toughness [141,142]. These techniques significantly enhance the tensile properties of hydrogel adhesives. To prevent hydrogels from flaking off during skin surface activity or stretching, the chemical composition, polymer content, molecular weight, and crosslink density need to be adjusted from the early stages of design to ensure a good match with the skin tissue.

When designing bionic hydrogel adhesives, it is crucial to consider their mechanical properties. The degree of matching between bionic adhesion materials and tissue adhesion materials will significantly affect their adhesion effect. If a mechanical mismatch exists, interfacial stress concentrations may lead to tissue damage or premature material failure. One solution is to introduce an elastic modulus gradient in the biomimetic adhesive so that the interface between the material and the tissue has a better mechanical match (i.e., softer), while the part covering the wound has a higher stiffness to resist deformation (i.e., harder) [143]. In addition, the rheological properties of bionic hydrogel adhesives are also critical to their adhesive properties, which determine their fluidity, ability to adapt to irregular tissue surfaces, and ability to form mechanical interlocks with uneven surfaces. In this regard, the viscosity of the bionic adhesive precursor should be carefully adjusted: overly viscous materials may not penetrate or conform to the tissue efficiently, whereas overly flowable materials may be washed away or form inadequate adhesion. Modulation of rheological properties, for example, through the addition of rheology modifiers, can optimize the bionic adhesive to ensure that it effectively covers and tightly conforms to irregular tissue surfaces, thereby improving adhesion performance [144]. In addition, the crosslinking kinetics of an adhesive are critical for its suitability in different clinical scenarios. Rapidly crosslinking adhesives are suitable for emergency situations and dynamic tissues, whereas a slower crosslinking process is appropriate for situations where precise application is required [137]. Adjusting the type of crosslinking agent, its concentration, and the availability of crosslinking sites allows for fine control of the reaction kinetics to meet different clinical needs.

## 5. Conclusions and Outlook

Bionic hydrogel adhesives hold great potential in skin wound therapy by exhibiting excellent biocompatibility and strong adhesive properties, while also overcoming many limitations of conventional adhesives, such as non-degradability, cytotoxicity, incompatibility with moist surfaces, and inability to adapt to dynamic tissue movement. Consequently, these bionic adhesives can be used to create innovative wound dressings and serve as medical glues to facilitate wound closure. Through bionic strategies, researchers have significantly improved the adsorption, mechanical interlocking, and electrostatic interactions of hydrogel adhesives to enhance adhesion properties. With functionalities such as self-healing, drug release, mechanical strength, and responsiveness, these adhesives can flexibly adapt to complex and changing wound environments, significantly accelerating wound healing.

However, due to the complexity and variability of the wound repair process, it is difficult to fulfill the needs of the entire healing process with a single dressing. For example, controlling inflammation is critical during the inflammatory phase but may not be needed during the subsequent repair phase. Additionally, the cells, cytokines, and functional components of a dressing usually only work at specific stages and may even have adverse effects at other stages. Therefore, providing function on demand has become a focus of further research. In addition, precisely controlling the degradation or separation of hydrogel adhesives from the skin surface when needed during practical application remains a challenge, especially for delicate and fragile skin, such as that of infants and patients with chronic wounds. Traditional adhesives are often difficult to control in real-time during the process of debonding and degradation, which limits their application flexibility and potential for personalized treatment. In the future, hydrogel adhesives will require a more sensitive response. Once their mechanical function is no longer needed (for example, when a skin wound has healed), the ideal hydrogel adhesives should be able to automatically trigger detachment or degradation. This triggering mechanism can be based on an external stimulus (such as a change in temperature or pH) or an intrinsic biological signal (such as the presence of a specific enzyme). With this design, the hydrogel adhesive can be easily removed without damaging the skin, thus reducing secondary damage and discomfort for patients. This unique personalized design may be one of the important directions for future adhesives. Chronic wounds are usually alkaline, but most pH response systems are better suited to acidic environments. Therefore, developing hydrogel adhesive dressings that can function effectively under alkaline conditions is of great significance. In the future, hydrogel adhesives will pay more attention to the development of personalized treatment, customizing hydrogel adhesives with exclusive functions and formulations for the specific conditions of patients to meet clinical needs. For example, for patients with chronic wounds requiring long-term care, such as diabetes patients, hydrogel adhesives with long-acting slow-release drugs could be designed to reduce the number and frequency of dressing changes. At the same time, this would respond to changes in blood glucose levels to enhance drug release and antibacterial function in a hyperglycemic environment. Combined with sensing technology, the status of wound healing could be monitored in real-time, allowing the function of the hydrogel adhesive to be adjusted based on this data, enabling precise treatment and timely detection of potential infection risks.

Although hydrogel adhesives have great potential in the treatment of skin wounds, there are still many shortcomings and challenges in the development of on-demand stripping, real-time control of the degradation process, personalized therapy, and integration with sensing technology. Future research and development should focus on these directions to further explore complex bionic bonding mechanisms and develop personalized hydrogel adhesives for different trauma environments to achieve safer, more effective, and personalized skin trauma treatment.
